# Viewing 3D TV over two months produces no discernible effects on balance, coordination or eyesight

**DOI:** 10.1080/00140139.2015.1114682

**Published:** 2016-01-13

**Authors:** Jenny C.A. Read, Alan Godfrey, Iwo Bohr, Jennifer Simonotto, Brook Galna, Tom V. Smulders

**Affiliations:** ^a^ Institute of Neuroscience, Newcastle University, Newcastle upon Tyne, UK; ^b^ Institute for Ageing and Health, Newcastle University, Clinical Ageing Research Unit, Newcastle upon Tyne, UK

**Keywords:** Stereoscopic displays, 3D television, stereo vision, binocular vision

## Abstract

With the rise in stereoscopic 3D media, there has been concern that viewing stereoscopic 3D (S3D) content could have long-term adverse effects, but little data are available. In the first study to address this, 28 households who did not currently own a 3D TV were given a new TV set, either S3D or 2D. The 116 members of these households all underwent tests of balance, coordination and eyesight, both before they received their new TV set, and after they had owned it for 2 months. We did not detect any changes which appeared to be associated with viewing 3D TV. We conclude that viewing 3D TV does not produce detectable effects on balance, coordination or eyesight over the timescale studied.

**Practitioner Summary**: Concern has been expressed over possible long-term effects of stereoscopic 3D (S3D). We looked for any changes in vision, balance and coordination associated with normal home S3D TV viewing in the 2 months after first acquiring a 3D TV. We find no evidence of any changes over this timescale.

## Introduction

Man-made visual images almost always contain information about the three-dimensional structure of the depicted scenes, whether through pictorial cues such as perspective or shading, or time-varying cues such as simulated motion parallax. However, stereoscopic information from binocular disparity is a particularly compelling depth cue, to the extent that displays containing this cue are often referred to simply as ‘3D’. We will follow this convention in this paper, using the term ‘stereoscopic 3D’, ‘S3D’ or simply ‘3D’ to refer to visual content containing binocular stereoscopic depth cues, and using ‘2D’ to refer to content lacking such cues.

S3D displays are nothing new. The first 3D feature film was made nearly 100 years ago (‘The Power of Love’, 1922). However, up till recently, stereoscopic 3D has generally been reserved for special ‘events’, whether an occasional feature film, a theme park ride or similar. Only in the last few years have advances in digital technology meant that stereoscopic 3D can become an everyday occurrence. Now, 3D televisions, Blu-ray recordings, smartphones and games systems mean that some users will be experiencing stereoscopic 3D on a daily basis, possibly for hours at a time.

This has led to concern in the media about possible long-term effects on viewers (e.g. ‘Keep doing that and you’ll go blind’, http://www.abc.net.au/unleashed/32814.html, retrieved 2 May 2013). Although there are now many scientific studies regarding S3D displays (for reviews, see (Lambooij et al. 2009; Tam et al. 2011; Urvoy, Barkowsky, and Le Callet 2013); all have assessed the immediate impact of isolated, brief exposure to S3D content: typically ranging from 5 min (Oliveira, Jorge, and González-Méijome 2012) to the length of a feature film (Read et al. 2015).

There is currently no data on possible adverse effects which could build up over the longer term with repeated, frequent exposure. Of course, such studies are difficult to carry out because they require long-term monitoring of large numbers of research participants.

In this paper, we report a first effort in this direction. In a previous paper (Read et al. 2015), we reported a typical, ‘acute’ lab-based study, in which 433 participants were tested on a range of visuomotor tasks both before and after viewing a movie in either 2D, active S3D or passive S3D. In the present paper, we report a follow-up study on a subset of 116 participants. Following the initial experiment, these participants were given a new high-definition television set to view as desired at home. Participants who viewed 2D TV in the lab were given a conventional 2D TV; those who viewed S3D content were given the same model of 3D TV. Over the following 2 months, participants were asked to report their screen use via daily online questionnaires, enabling us to assess how much conventional vs. stereoscopic content they were exposed to. These subjective reports were analysed and reported in a separate paper (Read 2014). The participants then came back to the lab to repeat the visuomotor tests. Each participant’s vision and binocular function was also assessed by two qualified eyecare professionals both before and after the study, using standard clinical tests. If viewing S3D television caused a measurable change in vision, depth perception or visuomotor function over the timescale of the study, we would expect to see a difference in the S3D groups compared to the 2D controls.

The lab-based visuomotor tasks and the clinical vision tests were both chosen based on the existing literature regarding short-term viewer experience with S3D. S3D content can cause adverse effects in some viewers, primarily eyestrain or headache (Emoto, Nojiri, and Okano 2004; Nojiri et al. 2004; Ukai and Howarth 2008; Lambooij et al. 2009; Howarth 2011; Yang et al. 2012; Solimini 2013; Read and Bohr 2014). The causes of the discomfort remain unclear. The most obvious reason is the vergence/accommodation conflict presented by S3D displays: that is, the difference between the vergence necessary to fixate the image with both eyes, and the accommodation necessary to bring the image into focus. In an S3D display, a virtual object may require vergence either in front of or behind the screen, while accommodation is always on the screen. Large mismatches between vergence and accommodation certainly produce visual fatigue and after-effects (Emoto, Nojiri, and Okano 2004; Emoto, Niida, and Okano 2005; Hoffman et al. 2008; MacKenzie et al. 2010; Shibata et al. 2011a; 2011b; Banks, Kim, and Shibata 2013), especially if the conflict changes rapidly (Jung et al. 2012; Kim, Kane, and Banks 2014), although commercial S3D content is designed to keep the vergence/accommodation conflict small and within viewers’ comfort zone.

S3D viewing is also often associated with motion sickness or dizziness (Yang and Sheedy 2011; Lee and Song 2012; Yang et al. 2012; Read and Bohr 2014). Samsung, a manufacturer of 3D TVs, refers in a warning notice to dizziness, lightheadedness, disorientation, decreased postural stability and risk of falling (http://www.samsung.com/ca/pdf/3D-tv-warning_en.pdf, retrieved 17 September 2015). A major contributing factor to motion sickness in simulators and other visual displays is thought to be the cue conflict between the visual image, indicating self-motion, and the vestibular system, indicating no motion (Lawson 2015). This may be heightened by the more realistic visual image in an S3D display. Additionally, in S3D displays the geometry is almost never veridical, potentially creating discrepancies in shape cues which could also contribute. Hands, Smulders, and Read (2015) showed that distortions in perceived shape due to oblique viewing were similar in S3D and 2D, but did not investigate any differences in comfort.

Given this background, we designed our lab-based tests to probe aspects of performance which might be expected to be most vulnerable to the adverse effects associated with S3D displays. As a test of visuomotor coordination, we asked participants to guide a hoop around a convoluted 3D track without touching it. This task requires good depth perception, and has been reported to benefit from good stereo vision (Murdoch, McGhee, and Glover 1991) and binocular viewing (Joy, Davis, and Buckley 2001; Read et al. 2013). Thus, we reasoned that any impairment of depth perception which could affect functioning in daily life should be detectable on this task. To detect any loss of balance due to S3D-induced dizziness, we had participants complete a short obstacle course while wearing two triaxial accelerometers. We assessed the time taken, errors made and postural stability as indicated by the accelerometry. In our previous paper, comparing performance on these tasks immediately before and immediately after viewing a movie, we could not detect any short-term effect of S3D (Read et al. 2015). In this paper, we now examine whether regular viewing of S3D over a period of 2 months produces any detectable change.

Some ophthalmologists have expressed concern that prolonged exposure to vergence/accommodation conflict in S3D displays could potentially contribute to visual disorders such as amblyopia (‘lazy eye’) (Pallas, Meyer, and Mojon 2013). We therefore paid particular attention to the possibility of changes in binocular visual function as assessed with standard clinical tests. To address this, a qualified orthoptist, i.e. an sightcare professional who usually works in a secondary clinical setting examining individuals with binocular vision disorders, tested each participant both before and after their 2 months with their new TV set. They carried out a set of standard clinical tests used in the diagnosis, characterisation and management of binocular visual disorders such as amblyopia and strabismus (‘squint’). These tests are designed to assess how well the two eyes work together, whether they have any tendency to become misaligned, the change in vergence induced by a given change in accommodation, the quality of stereoscopic vision, etc. They are all affected by binocular vision disorders such as strabismus and amblyopia, and are therefore well suited to detecting any change in binocular visual function associated with long-term viewing of S3D.

A binocular disorder such as amblyopia would also cause a loss of visual acuity in one eye. Problems with accommodation could also reduce acuity at particular distances. Some ophthalmologists have expressed concern that prolonged use of S3D displays could potentially contribute to visual impairment (Pallas, Meyer, and Mojon 2013). To address these concerns, a qualified optometrist, i.e. a sightcare professional who usually works in a primary care setting examining general eye health, measured each participant’s refractive error and their visual acuity in a range of conditions, including both near and far distances and while viewing with only one eye or both together. Together, this broad range of clinical tests was designed to reveal any adverse changes in either eyesight or binocular control which could be associated with stereoscopic 3D content over a two-month period. In combination with the lab-based tests of balance and visuomotor coordination, they represent a comprehensive assessment which should reveal any substantial deterioration associated with S3D.

## Methods

### Ethics

The study was approved by the Newcastle University Faculty of Medical Sciences Ethics Committee (approval number 00431) and adhered to the tenets of the Declaration of Helsinki. All participants, or in case of children, adults with parental responsibility, gave written informed consent. Year of birth and gender were reported by the participants. Details of recruitment methods are provided in Read (2014).

### Participants and recruitment

Participating households were given a new HD TV set to keep at home. They were asked to complete baseline questionnaires before participating, and also to complete brief daily online questionnaires on their screen use for 2 months (Read 2014). 10 households were given an active 3D TV using shutter glasses; these are referred to as the ‘A group’. 10 ‘B-group’ households were given a passive 3D TV using circularly polarising glasses. As a control group, a further 10 ‘C-group’ households were given a 2D TV. The TV models are specified in Table 1.

**Table 1.** Details of participants in the three different study groups. One C household dropped out of the study and one reported very large amounts of S3D screen time for all members; neither of these are included in the present analysis. ‘Age’ is actually year of birth subtracted from 2011 (year of the study).

TV-group	Code	A	B	C
	Meaning	Active 3D	Passive 3D	2D control
Model TV set provided		LG 47LX6900 with 4 pairs of AG-S100 active 3D shutter glasses	LG 47LD 920 with 6 pairs of adult and 6 pairs of children’s passive 3D glasses	LG 47LD450 with no 3D function
Number of households included in analysis		10	10	8
Number of participants (male/female)		41 (22/19)	42 (21/21)	33 (17/16)
Age (mean/median, SD, max-min)		27/21, 17, 4–59	30/24, 16, 7–67	27/22, 16, 4–49
Number of participants in age-group 1, ‘under-11s’ (birthyear 2001 or later)		9	5	7
Number of participants in age-group 2, ‘11–24’s (birthyear 1987–2000)		12	17	10
Number of participants in age-group 3, ‘30–40’s (birthyear 1971–1981)		8	5	5
Number of participants in age-group 4, ‘over-40’s (birthyear 1970 or earlier)		12	15	11

We initially recruited 30 households containing 125 participants. One C-group household dropped out of the study without giving a reason, shortly after completing the initial lab and eye tests. A second C-group household was removed from the data analysis based on their responses to the daily screen-use questionnaires. For this study, it is crucial that the so-called S3D groups, A and B, do indeed watch more S3D content than the 2D control group C. This C-group household, H83C, reported very large amounts of S3D screen time (>5 h a day). If some of the C-group participants were watching large amounts of 3D TV, this could make our 2D and S3D groups more similar and risk obscuring a genuine effect of S3D. Although there were reasons to doubt the validity of H83C’s reports (Read 2014); we excluded this household from the analysis presented in this paper. Thus, this paper presents results from 116 participants in 28 households (Table 1).

### S3D exposure score

We used the online questionnaires to define a ‘S3D exposure score’ estimating how much S3D content each participant viewed during the study. In the online questionnaires, participants reported time spent viewing 3D TV, cinema and video games by selecting, for each type of content, one of the following responses: ‘less than 60 min’, ‘1–2 h’, ‘2–3 h’, ‘3–5 h’ and ‘more than 5 h’. To construct our measure, we assigned these 5 responses a score from 0 to 4, respectively. For each content type, we calculated the mean score averaged across all the questionnaires completed by that participant, and summed over content type. This resulted in a S3D exposure score ranging from 0 (for participants who never reported viewing any 3D content) to a theoretical maximum of 12 (if a participant had reported viewing 5 h of 3D TV, 5 h of 3D cinema and 5 h of 3D games on every questionnaire completed).

### Tests carried out

Before and after the 2 months of owning their HD TV, participants visited a local optometry practice where their eyes and vision were examined by qualified optometrists and orthoptists. Test protocols are described in detail in Read and Bohr (2014) and Read et al. (2015).

For participants aged eight and over, visual acuity was measured at 0.4 and 6 m for both monocular and binocular viewing, using the best optical correction as determined by a previous measurement of refractive error. At 6 m, acuity was also measured using the participant’s habitual correction, resulting in a total of nine separate acuity measurements at each visit. For participants aged seven and under, monocular and binocular acuity was measured at 3 m with the participant’s habitual correction, for a total of three acuity measurements.

Single clear binocular vision requires a person to direct both eyes at the object of interest so that it falls on the foveas (convergence) and also to focus their eyes on it so that it appears sharp (accommodation). These adjustments are driven by the retinal image, and are also neurally cross-linked so that a change in accommodation automatically triggers a change in convergence and vice versa (Schor 1992). Good stereoscopic vision additionally requires binocular neurons in visual cortex. The orthoptists assessed all these aspects of binocular visual function using several standard clinical tests. Stereoacuity, i.e. the smallest binocular disparity difference the participant can reliably detect, was measured with two tests which are widely used in UK clinical practice: the Frisby test at near distances (30–80 cm) and with the revised FD2 test at distance (6 m). Both these are real-depth tests which measure a patient’s ability to identify a test object as being closer than reference objects. In the Frisby stereotest, the objects are patterns printed on either side of a plastic plate; the plate thickness, together with viewing distance, controls task difficulty. In the FD2 stereotest, the objects are geometric shapes presented within a square box. The shapes are on rods which allow the test shape to be pushed physically closer.

**Figure 1.**
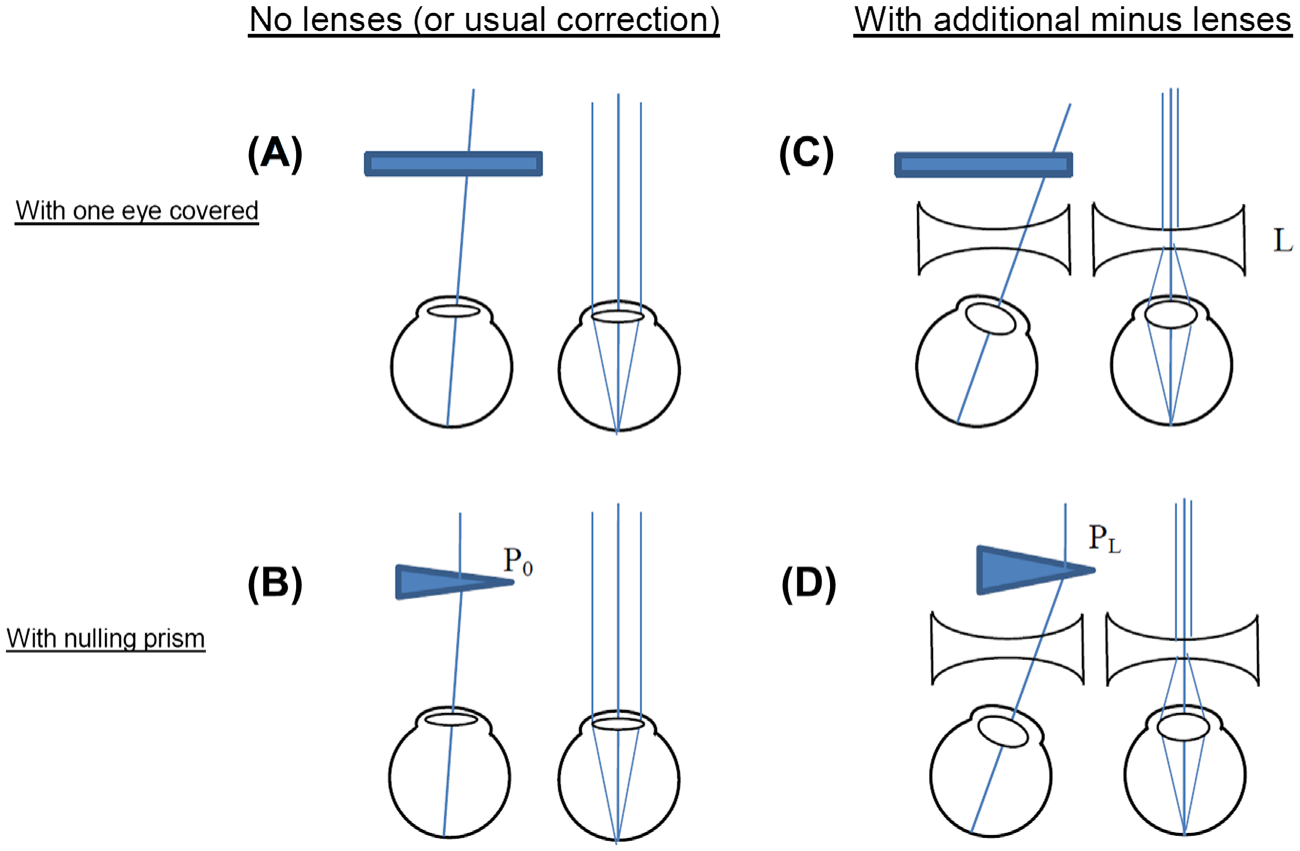
Gradient method for measuring AC/A ratio. The eyes view an object at 6 m (effectively infinity), so accommodation is relaxed. One eye is covered (A). It then takes up the convergence state which is neurally hard-wired for this accommodation state, i.e. the accommodative convergence. This is measured by finding the strength, *P*_0_, of the prism which is required in order to cancel out any movement when the eye is covered and then uncovered (B). This prism enables both eyes to fixate the object while keeping their natural accommodative convergence. This process is then repeated with diverging lenses (minus lenses) in front of the eyes (C and D); L is the strength of the diverging lens, in dioptres.

Notes: The diverging lenses force the eyes to accommodate more strongly in order to keep the target in focus. Because of the neural coupling between accommodation and convergence, this means that the eyes spontaneously adopt a more converged state when one eye is covered. This is again measured by finding the strength, *P*_L_, of the nulling prism (D). The AC/A ratio is given by the difference in accommodative convergence divided by the difference in accommodative demand, i.e. AC/A = (*P*_L_−*P*_0_)/|L|.

Near point of convergence (the minimum distance on which participants can both converge and focus their eyes, without experiencing blurred or double vision) was measured with a RAF rule. The accommodative-convergence to accommodation (AC/A) ratio was measured with the gradient method. The AC/A ratio quantifies the neural coupling between accommodation and convergence. It does so by measuring the change in convergence produced by a change in accommodation, when one eye covered so that there is no binocular cue to convergence (see Figure 1 and legend).

In addition, before and after the 2 months, participants visited Newcastle University Institute of Neuroscience and carried out a set of tasks designed to test balance and coordination. These tests are detailed in Read et al. (2015). Briefly, the coordination test required participants to guide a wire hoop around a complicated 3D track without making contact. Accuracy was assessed by recording the voltage changes which occurred when the hoop touched the wire; timing was also recorded electronically. The balance test required participants to walk over a ramp, along a low beam and step over foam blocks. Pressure-sensitive mats in the floor recorded their timing, and errors such as stepping off the beam, while tri-axial accelerometers at hip and chest enabled aspects of their postural stability and gait to be assessed.

After carrying out these balance and coordination tests, participants viewed a movie, and then carried out the tests again. Participants viewed the movie in 2D or 3D according to their group. That is, the A-group viewed the movie in 3D on an active 3D TV set with shutter glasses; the B-group viewed the movie in 3D on a passive 3D TV set; and the C-group viewed the movie in 2D. In this paper, we will refer to the tests before and after viewing the movie as different *sessions*. Thus, the balance and coordination tests were carried out four times: in two *sessions* (before vs. after viewing the movie) on each of two *visits* 2 months apart. The eye tests were carried out twice, in two visits 2 months apart. On the first visit, participants watched the movie ‘Toy Story’ in either 2D or 3D according to their group; on their second visit, they watched ‘Toy Story 2’, again in either 2D or 3D.

### Data analysis

Visual acuity data were roughly normally distributed, so we analysed them using a repeated measures ANOVA with visit, eye and test condition as the within-subject factors, and TV-group the between-subject factor. ANOVA is robust to small departures from normality. Stereoacuity and other eye-test data were highly non-normal. To analyse these, we first computed the change in each participant’s score between the first and second visits, and looked for an effect of TV-group on these differences using the non-parametric Kruskal–Wallis analysis of variance by ranks.

We analysed the balance and coordination data using a repeated measures ANOVA with visit and session as the within-subject factors, and TV-group and age as the between-subject factors. Because the number of participants within each TV-group was not large enough to include age as a continuous variable, for this analysis we grouped participants into four discrete age groups, given in Table 1

We examined the short-term effects of viewing a 3D movie in a previous paper with a larger group (Read et al. 2015). Our randomisation procedure was designed to remove a main effect of TV-group, although with only ~10 households in each group, this was not always successful. Any main effect of session or visit likely reflects the effect of practice or familiarity with the tests. Main effects or interactions involving age are also not surprising, but not our concern here. Our specific research question, regarding the effect of viewing 3D TV, is revealed in the interaction terms which include TV-group. In our previous paper with a larger cohort (Read et al. 2015), we studied any short-term effects which viewing a 3D movie might have on balance and coordination. These would here be reflected in the interaction between TV-group and session. In this paper with the smaller follow-up group, we are mainly interested in any long-term changes detectable over the 2 months of the study. This is revealed in the interaction between TV-group and visit.

The methods used to analyse the accelerometry data were described previously (Read et al. 2015). Briefly, we low-pass filtered the accelerations to remove noise. We calculated the standard deviation of the accelerations recorded while the participant performed each of the three balance tasks (ramp, beam, steps), and used this SD as a metric of body motion during the task.

### Power calculation

We calculated the minimum effect size that we had the power to detect. In this study, we have compared several different parameters on two different visits 8 weeks apart. In each case, our null hypothesis is that TV-group (3D-active, 3D-passive or 2D) has no effect on the change *x* under consideration, i.e. that the mean change between visits is the same for all three groups. We calculated the effect size we would require in order to achieve a power *π* = 0.8. Given that the mean sample size in each of our groups is 39, the minimum detectable effect size is about *f* = 0.3, where *f* ^2^ is the ratio of the variance of the group means to the variance within the groups. Assuming intermediate variance between the different TV groups, the smallest range in group means we would expect to detect would be 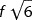 = 0.7 (Cohen 1988). That is, our study has enough power for us to reliably detect an effect of 0.7 of the within-group standard deviations.

## Results

### S3D exposure score

**Figure 2.**
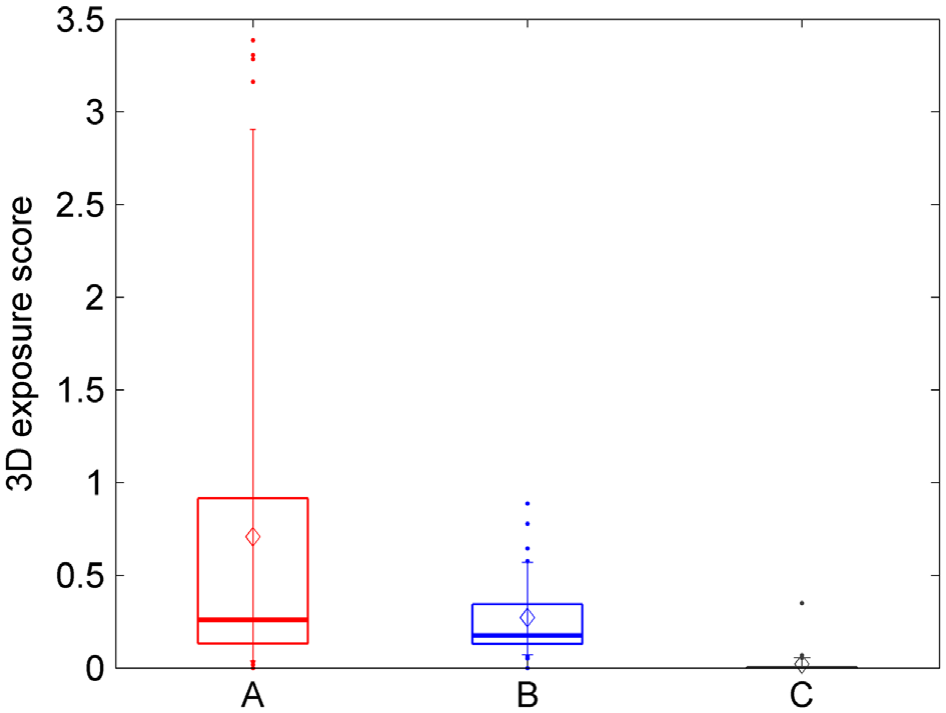
S3D exposure prior to study. Box and whisker plot for S3D exposure score (see Methods), for each of the 3 TV-groups specified in Table 1.

Notes: Thick lines show the median; diamonds show the mean; boxes run from 25 to 75% percentile; whiskers run from 9 to 91% percentile; dots show individual data-points outside these limits. The minimum score in each group was 0 (no 3D content viewed); the maximum was 3.4 for A-group, 0.9 for B and 0.4 for C; the medians were 0.26, 0.18, and 0 respectively.

Figure 2 shows the mean S3D exposure score (TV, games and cinema) for participants in the different groups. Recall that S3D exposure score measures the average amount of S3D which a participant reported viewing per day, where a value of 0 indicates ‘<60 min’, 1 means ‘1–2 h’ and 3 means ‘3–5 h’. A-group and B-group watched much more 3D content than the C-group (Kruskal–Wallis test, *χ* ^2^(2) = 57.84, *p* < 0.001; pairwise tests show no difference between A and B: Mann–Whitney *Z* = 1.23, *p* = 0.219, but significant differences with C: A v. C: *Z* = −6.62, *p* < 0.001; B v. C: *Z* = −6.89, *p* < 0.001). As expected, this mainly reflected differences in the amount of 3D TV viewed. The A and B groups, who were given a new 3D TV in their home, watched substantially more 3D TV than the C group, who were given a 2D TV. 3D cinema and video/computer games contributed very little. The mean cinema and game scores were zero, to one decimal place, for all three groups. The questionnaires completed at recruitment indicated that the great majority of participants viewed S3D only ‘a few times a year’; the five households who reported viewing 3D TV ‘once a month’ at recruitment were distributed evenly across the different TV-groups (Read 2014). This analysis indicates that our experimental manipulation was successful, i.e. giving people a 3D TV did increase their exposure to S3D content. Even within the 3D groups, however, participants varied widely in their amount of S3D exposure during the study. In the analysis below, we will correlate changes with the S3D exposure score, in order to assess whether 3D viewing can be associated with any of these changes.

### Eye tests

We examined our data to look for evidence of changes in visual function which could be associated with 3D TV.

#### Visual acuity

Visual acuity is the most fundamental metric of eyesight. Any change in visual acuity associated with 3D TV would be of great concern. Potentially, any changes might affect acuity in some circumstances but not others. For example, a reduction in the accommodative response might impair acuity at only one distance, or an improvement in binocular function might increase the advantage of binocular viewing. We therefore made several different measurements of acuity, including viewing with one eye or with both, and at near or far distance, as described in the Methods.

**Figure 3.**
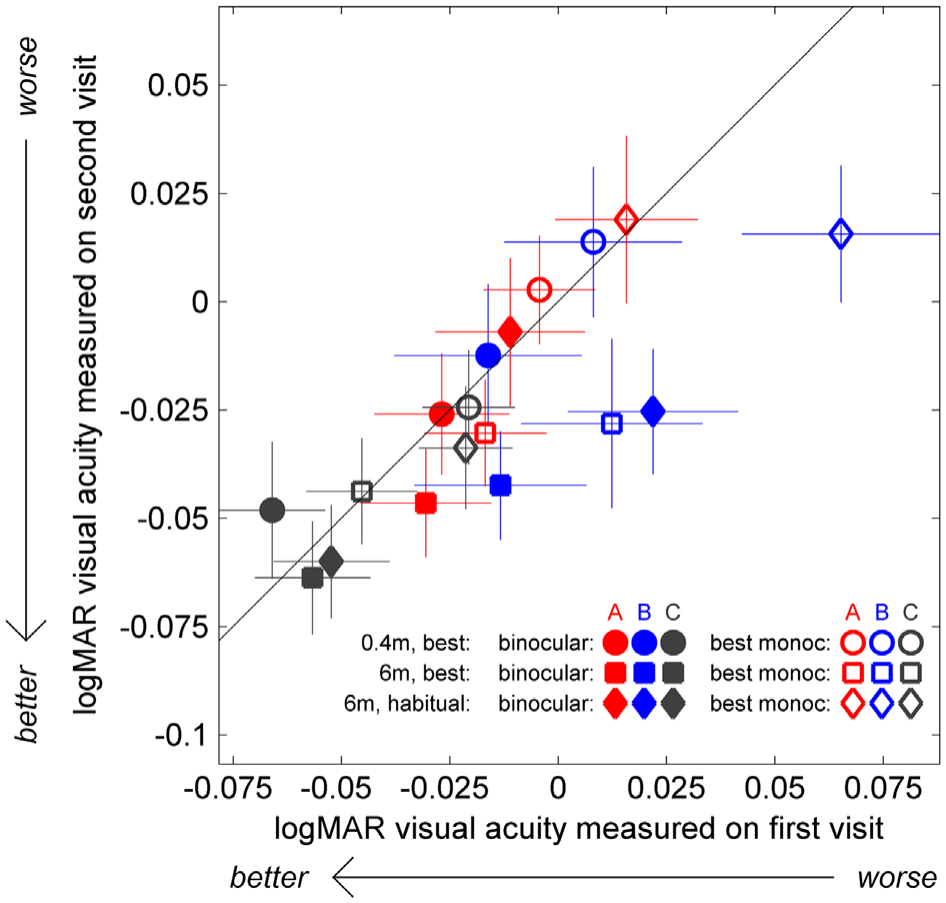
Change in visual acuity.

Notes: Data points show logMAR binocular visual acuity on first and second visits, i.e. before and after possessing the TV at home for 2 months. A higher logMAR score indicates worse vision; a logMAR score of 0.0 corresponds to 20/20 vision. Colours show the three TV-groups; symbols show the visual acuity test condition (viewing distance, either 0.4 or 6 m, and optical correction, either best or habitual). Filled symbols show visual acuity measured with binocular viewing; open symbols show the best of the two visual acuities measured monocularly. Symbols show mean acuity over all group members for whom data were available (excluding under-8s); error bars show ±1 standard error on the mean.

Acuity data were available for a total of 108 participants (37 A, 41 B and 30 C). The results are complicated by six participants who did not wear an optical correction at the initial testing, but did at the second testing (2 A-group, 2 B, 2 C). In some cases, this was because their need for optical correction was picked up at the initial exam. In accordance with study ethics, in this case, participants were recommended to visit an optometrist. We analysed the data both with and without these six participants. Figure 3 compares visual acuity, in logMAR, on the first and second visits. Note that better acuity corresponds to smaller logMAR scores.

We ran a mixed-design ANOVA with visual acuity as the dependent variable, TV-group as the between-subject factor and three within-subject factors: (1) ‘eye’, comparing results when tested with both eyes vs. with the best-acuity eye only; filled vs. open symbols in Figure 3; (2) ‘test’, i.e. at 0.4 m with best correction (symbol ● in Figure 3), at 6 m with best correction (■) or at 6 m with habitual correction (♦); and (3) ‘visit’, i.e. first visit vs. second visit. As expected, we found that people had better acuity with their best correction than with their habitual correction (and better at 6 m than at 0.4 m; *F*(2,104) = 15.125, *p* < 0.001), and also that they performed better with two eyes than with their best eye monocularly (*F*(1,105) = 116.68, *p* < 0.001). We did not find an overall effect of visit (*F*(1,105) = 2.72, *p* = 0.102). Measured acuity improved slightly from the first to the second visit on tests at 6 m, but not on the test at 0.4 m (Interaction: *F*(2,104) = 4.34, *p* = 0.015). This effect is visible in Figure 3: the circles generally lie on or above the identity line, indicating similar or worse acuity at 0.4 m on the second visit, whereas squares and diamonds generally lie below, indicating better acuity on the second visit. This probably represents a practice effect, which may be greater at 6 m because people are less used to reading at this distance. The only other significant effect we found was an interaction between ‘eye’ and ‘test’ (when viewing with the best-acuity eye only, the differences between the three test conditions were all significant, while when viewing binocularly, the only significant difference was at 6 m comparing best vs. habitual optical correction; Interaction: *F*(2,104) = 4.87, *p* = 0.009). None of these results changed significantly if we left out the six individuals who acquired an optical correction between the first and the second visit.

The main effect of TV-group approached significance (*F*(2,105) = 2.72, *p* = 0.07; *F*(2,99) = 3.47; *p* = 0.035 without the 6 individuals who acquired correction), with the control group C having higher acuity than the other two groups. Again, this is visible in Figure 3: grey symbols are more negative than coloured symbols. However, this difference existed already at the start of the study and cannot therefore be attributed to the effects of viewing 3D TV. As described in the Methods, we did not match the three groups on visual acuity (or anything else) at recruitment, but assigned households to groups at random. With only 10 households in each group, some differences evidently survived this randomisation procedure.

Critically, TV-group did not show any significant interactions with any of the measures, including visit (all *p* > 0.171). This indicates that whether participants viewed 3D or 2D TV had no effect on their visual acuity. Similarly, there was no correlation between change in acuity and S3D exposure (e.g. for binocular visual acuity with habitual correction at 6 m, the Pearson correlation is *r* = 0.136, *p* = 0.161, Spearman *r* = −0.052, *p* = 0.596, for 108 participants who performed this test on both visits.). As Figure 3 shows, if anything, the passive 3D group showed a greater improvement in acuity than the other groups.

#### Stereoacuity

**Figure 4.**
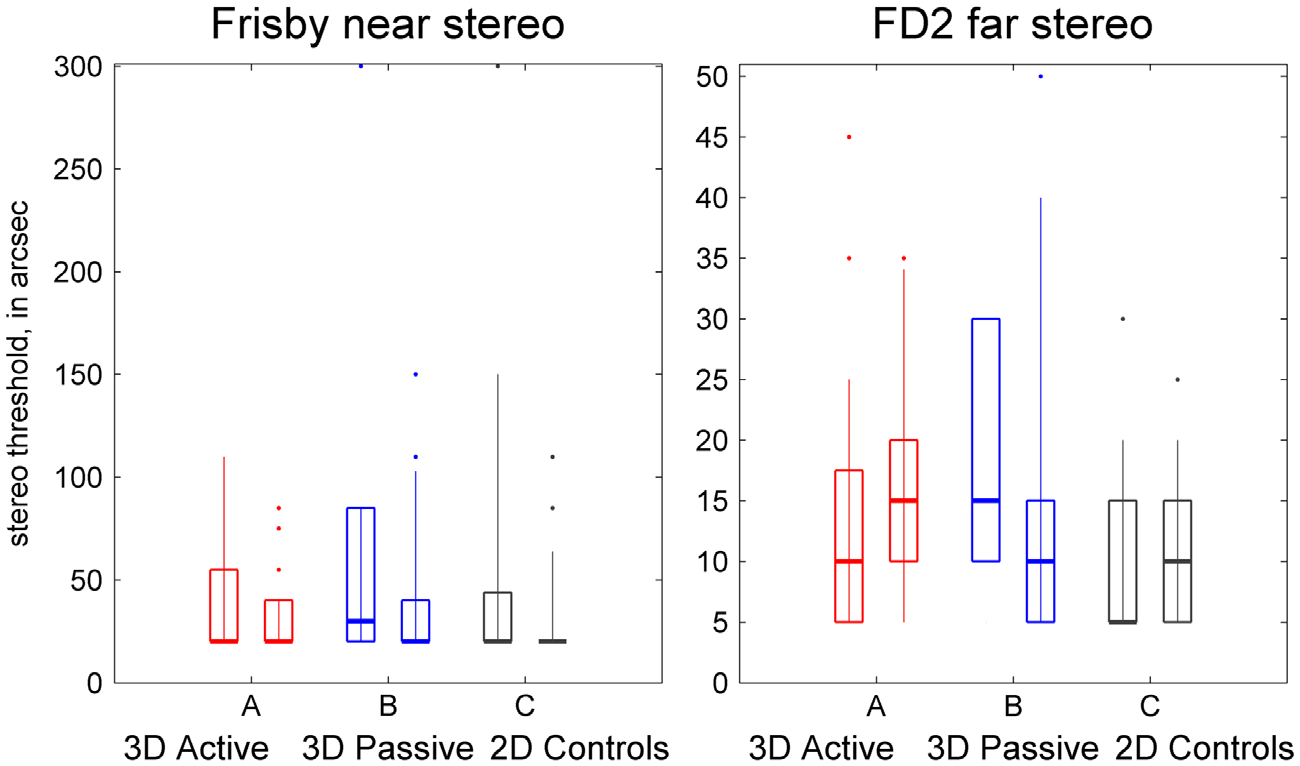
Changes in stereoacuity.

Notes: Box-and-whisker plots of stereo thresholds measured with the Frisby and FD2 tests, for the three different TV-groups before (left box in each pair) and after (right boxes) the 8-week study. A higher threshold indicates worse stereo vision. Symbols and error bars as in Figure 2. Outlier dots do not show five stereonegative participants.

Stereoacuity was assessed using the Frisby stereo test for near viewing, and the Frisby-Davis distance stereo test at a distance of 6 m. The relationship between stereoacuity measurements with the two tests is discussed in Bohr and Read (2013). Although Frisby stereo thresholds measured on the two visits were highly correlated (Spearman correlation *r* = 0.44, *p* < 10^−6^), there was a significant improvement in stereoacuity across the two visits (*p* < 0.001, Wilcoxon signed-rank test; Figure 4). Averaged over all 116 participants, the mean reduction in stereo thresholds was 13 arcmin on the Frisby test. There was no difference in improvement between TV groups (Kruskal–Wallis test, *χ* ^2^(2) = 1.17, *p* = 0.56). Thus, the improvement is likely to be due simply to practice or familiarisation with the test.

The Frisby test as used in this study permits disparities from 20 up to 600 arcsec to be displayed (Bohr and Read 2013). Only one participant tested stereonegative on the Frisby test (i.e. could not perform the task at the largest disparity, despite demonstrating understanding of the task). This participant tested stereonegative on both visits, so the change in measured stereoacuity was zero. The FD2 test at 6 m permits disparities from 5 to only 50 arcsec, so people are more likely to test stereonegative on the FD2 test (Bohr and Read 2013). In our data-set, five people were FD2-stereonegative on the first visit, and four on the second, only one of whom was stereonegative on both. Additionally, we had to exclude one four-year-old A-group participant who could not be tested on the FD2 at the first visit due to poor cooperation. The FD2 is harder than the Frisby for children, probably because it is harder to direct their attention to a distant target (Bohr and Read 2013).

On the FD2 test, quantitative measurements of change in stereo threshold ranged between ±45 arcsec (if a participant scored 5 arcsec on one visit and 50 arcsec on the other). The six participants who were FD2-stereonegative on only one visit could not be assigned a quantitative measurement of change in stereo threshold. For purposes of analysis, we took the change to be ‘above 45 arcsec’, and analysed the data using techniques which depend only on the ordinal values.

Unlike the Frisby, there was no change in FD2 stereoacuity between visits when considering all participants together (*p* = 0.70, Wilcoxon signed-rank test). However, there was a significant difference between TV-groups (*χ* ^2^(2) = 18.3, *p* < 0.001, Kruskal–Wallis test). The 40 A-group participants showed no change (median 0), the 42 B-group participants showed a median improvement of 5 arcsec, while the 33 C-group participants showed a median decrease in performance (median increase in stereo threshold was 5 arcsec). The B-group differed significantly from both the A and C groups (*p* < 0.001 for both; Mann–Whitney *U* test) for change in FD2 stereo thresholds, although not in the Frisby.

Several lines of evidence suggest that this difference between TV-groups is due to random variation in the samples, rather than because viewing 3D TV really does improve stereoacuity. First, the B-group also differed in FD2 stereoacuity at recruitment (Kolmogorov–Smirnov test as above; *p* = 0.05, B vs. A; *p* = 0.012, B vs. C). Only five participants tested stereonegative on the FD2 at the first visit, and these were all in the B-group, three of them in the same household. Because the B-group happened to perform slightly worse on the initial visit, they had more room for improvement on the second visit, and this may well explain the difference. Second, the fact that stereoacuity improved only in the B group when tested with the FD2 (not in the A group and not when measured with the Frisby test), makes it less plausible that it is due to a genuine effect of TV type. Of course, it is possible that distance (but not near) stereoacuity is improved by viewing passive (but not active) 3D, but the effect would certainly be more convincing if it showed up in different data-sets. Finally, there was no correlation between S3D exposure score and change in FD2 stereo threshold, either for all participants (Spearman rank correlation *ρ =* −0.15, *p* = 0.12) or for the 42 B-group participants (*ρ =* −0.09, *p* = 0.56). Thus, our study does not provide convincing evidence that viewing 3D TV can improve stereoacuity. More importantly, there is no evidence at all that it has any adverse effect on stereoacuity.

#### Eye movement control

In natural viewing, there is a strict relationship between the vergence required to fixate an object and the accommodation required to focus on it. S3D displays depart from this natural relationship. When viewing an S3D screen, the viewer must always accommodate on the screen, while screen parallax may require them to converge either in front of or behind the screen. There have been some reports of short-term changes in accommodation response after viewing S3D content (Yano and Emoto 2002; Yano et al. 2002; Yano, Emoto, and Mitsuhashi 2004). It is therefore important to examine whether habitual viewing of S3D TV in a normal home environment produces any detectable changes in eye movement control.

The orthoptists assessed participants for latent and manifest abnormalities of eye movement, known, respectively, as phoria and tropia, using the cover test. In a tropia, or manifest deviation, the eyes are misaligned so that they cannot both fixate the intended object. A tropia may be either constant or intermittent. In a phoria, or latent deviation, the eyes usually both succeed in fixating the intended object when this is visible to both eyes, but they become misaligned when one eye is covered. In the cover test, the participant is asked to fixate an object, first with both eyes uncovered and then with one covered. If the participant has a tropia, one eye will not successfully fixate the object even when both eyes are uncovered. When the fixating eye is then covered, the other eye will now visibly turn inwards or outwards to acquire fixation. If the participant has a phoria, they will be able to fixate the object when it is visible to both eyes. However, when one eye is covered, the other eye will then drift out of position. When the cover is removed, the phoria will be visible as a movement of the other eye back into fixation. Horizontal and vertical phoria/tropia was assessed at 0.3 m and at 6 m. For participants with a non-zero phoria or tropia, the orthoptists quantified the deviation using prism bars to null the ocular deviation. The orthoptists also qualitatively examined ocular motility for any obvious abnormalities such as those associated with sixth nerve palsy, by asking participants to look in various directions. On the first examination, 8/116 participants were noted to have some abnormality of ocular motility and/or head posture; six of these also had phoria or tropia. On the second examination 2 months later, only three of these eight participants were again noted as having abnormal ocular motility/head posture, all in the B-group, while a further two participants were now noted as abnormal, again both in the B-group. However, due to the very low numbers involved, the differences between TV-groups were not statistically significant.

To examine whether control of binocular eye movements declined for participants in the S3D groups, we first used the qualitative results of the cover tests. For each visit, we assigned each participant a ‘binocular deviation score’ based on the worst score in these four conditions. A score of 0 indicated no deviation observed for either direction or distance. A score of 1 indicated a phoria but no tropia. A score of 2 indicated intermittent tropia, while a score of 3 indicated a manifest tropia. We then examined whether participants’ scores were worse on the second visit than on the first. 73/116 (63%) scored the same on both occasions, including 47/116 (41%) who had no observable deviation on either visit. Twenty-six participants scored 1 point worse and 17 scored 1 point better. The scores on first and second visits were not significantly different (*p* = 0.17, Wilcoxon signed rank test). There was no effect of TV-group (*p* = 0.25, Kruskal–Wallis test on difference score with TV-group as the factor). There was no correlation between S3D exposure and the change in binocular deviation score (the Pearson correlation *r* = −0.04, *p* = 0.69; Spearman *r* = −0.03, *p* = 0.75).

As noted above, phoria and tropia were also measured quantitatively in prism dioptres for all participants. Negative value indicated exodeviation, i.e. an eye turning outwards (‘wall-eye’), and positive indicated esodeviation, i.e. an eye turning inwards (‘cross-eye’); zero indicated no deviation. We looked at the difference in this measured deviation between the first and second visits. A process which made people more likely to exodeviate, say, would show up as a change in the mean of this metric. However, an effect which tended to increase both exotropia and esotropia (i.e. made a negative deviation more negative and a positive deviation more positive) would change the variance but not the mean. We therefore also looked at the difference between the absolute deviations. We did these analyses for both vertical and horizontal deviations, measured at both 33 cm and 6 m. There was no difference between TV-groups in any condition (Kruskal–Wallis test with TV-group as the factor; *p* > 0.26 for all 8 tests). There was also no correlation between S3D exposure and difference or absolute difference in deviation, by either the Pearson or Spearman tests (*p* > 0.25 for all 16 correlations).

Orthoptists also measured participants’ near point of convergence, representing the closest object a person can see while keeping both eyes in focus and aligned on the object. There were no significant changes in near point of convergence, either between visits (Wilcoxon signed rank test, *N* = 116, *p* = 0.84) or between TV groups (Kruskal–Wallis test, *χ* ^2^(2) = 1.67; *p* = 0.43). Measurements on the two visits were highly correlated ( the Pearson correlation *r* = 0.5, *p* < 10^−7^). There was no correlation between S3D exposure score and change in near-point ( the Pearson correlation *r* = −0.012, *p* = 0.9; Spearman *r* = 0.066, *p* = 0.48).

Finally, orthoptists measured accommodative convergence to accommodation (AC/A) ratio using the gradient method (Figure 1). The AC/A ratio measures the change in convergence evoked by a given change in accommodation, and assesses the strength of the neural relationship between convergence and accommodation. The values measured by our orthoptists were surprisingly low. Normal values are usually said to be in the range of 2–5, but the mean value amongst our sample was 0.6 on the first test and 1.0 on the second, with several negative values (i.e. where increasing the accommodative demand apparently reduced convergence). This may be because the orthoptists did not use objective measures of accommodation, and the participants may have had difficulty reporting their accommodation state accurately. The AC/A ratio measured on the first visit was particularly low in the A and C groups (mean 0.3 and 0.4 respectively, compared to 1.1 for the B group). This difference between groups at recruitment was highly significant (Kruskal–Wallis test, *χ* ^2^(2) = 25.2; *p* < 10^−5^), and represents a failure of randomisation due to our limited sampling. The AC/A ratio increased significantly between the first and second visits in both the A and C groups, so that by the second visit there was no significant difference between TV groups (Kruskal–Wallis, *χ* ^2^(2) = 0.04; *p* = 0.98). There was, therefore, a highly significant effect of TV-group on the *change* in AC/A ratio (Kruskal–Wallis, *χ* ^2^(2) = 19.1; *p* < 10^−4^). Because this increase occurred in both the A and C groups, it is not likely to be related to viewing 3D television.

**Figure 5.**
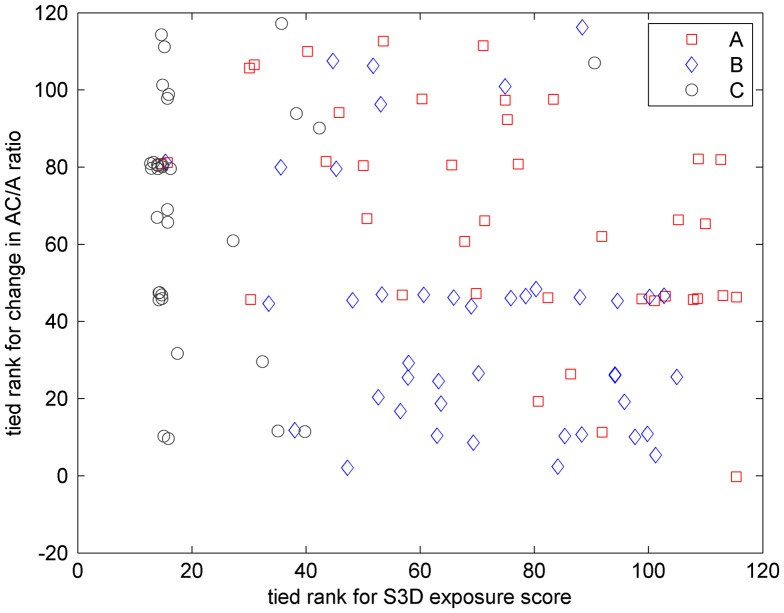
Relationship between S3D exposure and change in AC/A ratio between the two visits.

Notes: The Pearson correlation between the two was not significant, but the Spearman correlation (equal to the Pearson correlation between the tied ranks) was significant (*p* = 0.004). For this reason, we here plot the tied ranks of the data. Symbols have been jittered to avoid obscuring each other, e.g. without jitter, the 12 points at (14.5, 80) would coincide.

When we relate change in AC/A ratio to S3D exposure, we do find a weak inverse relation. The Pearson correlation between S3D exposure score and change in AC/A ratio was not significant (*ρ* = −0.11, *p* = 0.24), but the Spearman correlation was significant (*ρ* = −0.27, *p* = 0.004). This is partly driven by the large number of C-group participants who watched no 3D content and, as noted, generally showed an increase in AC/A ratio, whereas B-group participants tended to watch relatively large amounts of S3D content and show relatively small changes in AC/A ratio. However, even when members of the 2D control C-group are excluded, there is still a fairly significant Spearman correlation between S3D exposure and change in AC/A ratio (*N* = 83, *ρ* = −0.25, *p* = 0.02). That is, while AC/A ratio generally increased between the two visits, this increase tended to be less for people who viewed more S3D content. Potentially, this could indicate that viewing S3D tends to weaken the neural relationship between accommodation and convergence. The reasoning would be that because S3D stimulates changes in convergence without the corresponding change in accommodation, convergence and accommodation become somewhat decoupled, so that an increase in accommodation stimulates less convergence in response. However, as Figure 5 shows, the relationship is hardly compelling. Any relationship between change in AC/A ratio and S3D exposure is very weak (much less than the random variation between groups at recruitment, for example). We have performed multiple different statistical tests investigating whether orthoptic measures such as phoria/tropia, near-point, Frisby and FD2 stereoacuity as well as AC/A ratio are affected by viewing S3D. By definition, ‘significant’ results will occur on 5% of these tests by random chance, even under the null hypothesis that there is no relationship. If we apply the Šidák correction for multiple comparisons with five tests, the significance level becomes 0.01. At this adjusted threshold, the relationship between AC/A ratio and S3D exposure is not significant, and it is therefore likely that this is a spurious relationship. The experiment would need to be replicated, ideally with a larger sample, to be sure that it is indeed the case.

### Coordination test

The coordination test required participants to guide a wire hoop around a wire track without bringing the two into contact. This task benefits particularly strongly from binocular vision (Joy, Davis, and Buckley 2001; Read et al. 2013), and optimal performance may require stereopsis (Murdoch, McGhee, and Glover 1991).

In order to ensure suitable task difficulty for participants ranging from 4 to 83 years old, three different tracks of different complexity were available, and participants could choose which to complete. Seventy-two per cent (83/116) completed the same track on both visits. Only these participants were used in analysis.

**Figure 6.**
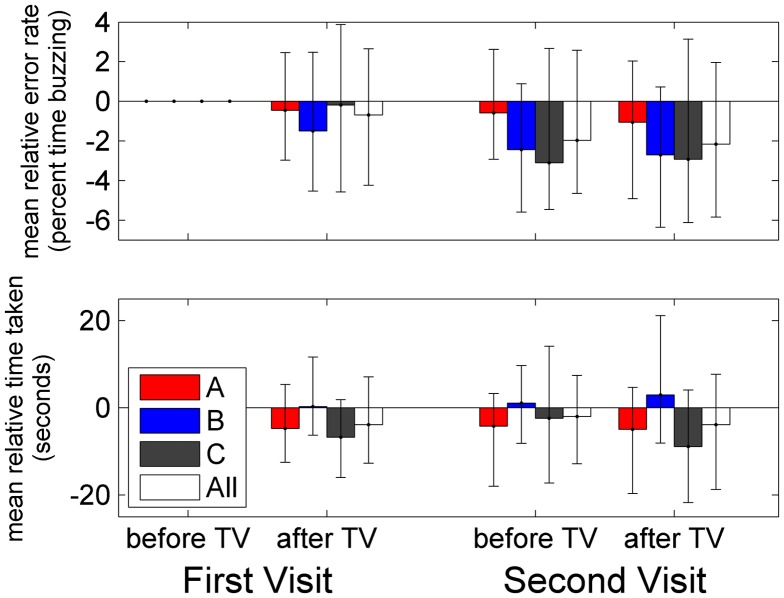
Visuomotor coordination.

Notes: How performance on the coordination task varied on the four occasions of testing, i.e. before and after viewing a movie on TV, on the first visit and on a second visit after having had a new TV for around 2 months. Performance is assessed with two metrics, corresponding to accuracy and speed: (A) error rate, i.e. percentage of this time spent with the loop in contact with the track, and (B) time taken to complete the task. Both metrics are shown relative to performance the first time it was measured; the ‘First Visit, before TV’ results are therefore zero by definition for all groups. Results are shown only for the 83/116 participants who completed the same track on all four occasions. Coloured bars show results for the different TV-groups; white bars show results for all 83 participants. Height of bars show mean change in metric averaged across participants; error-bars run from the 16th to the 84th percentile.

Figure 6 shows how performance on the coordination task varied on the four occasions of testing. The upper panel shows total time taken to complete the task, and the bottom panel shows the percentage of this time of which the hoop was in contact with the wire. To help remove between-subjects variation, we show these relative to the results at first test. Data are shown for the three different TV-groups (coloured bars) and averaged over all 83 participants (white bars). Unsurprisingly, most participants are better on both metrics in the second session, after viewing TV on their first visit. This improvement largely persists on the second visit.

For these 83 participants, we ran ANOVA with visit and session (before/after TV) as within-subjects factors, and TV-group and age-group as between-subjects factors. We first examined the errors made on the task, defined as the percentage of time for which the hoop was in contact with the wire (Figure 6(A)). Previous results suggest that accuracy on this task benefits particularly strongly from binocular vision (Read et al. 2013) and good stereoacuity (Murdoch, McGhee, and Glover 1991). Here, there was a main effect of visit (*F*(1,71) = 9.99, *p* = 0.002) but not of session (*F*(1,71) = 1.53, *p* = 0.220). There were marginally significant interactions between visit and age (visit*age-group: *F*(3,71) = 2.89, *p* = 0.041) and visit, age and TV-group (visit*age-group*TV-group: *F*(6,71) = 2.30, *p* = 0.044). Both these interactions became non-significant when we removed a single child in the 2D group, participant H164C005, who showed a particularly large improvement between visits (from >50% error to <10%). With H164C005 removed, these interactions are age*visit: *F*(3,70) = 1.353, *p* = 0.264; age*visit*TVgroup: *F*(6,70) = 0.803, *p* = 0.571. With or without H164C005, there was no interaction between TV-group and either visit or session. Thus, viewing 3D TV does not seem to be associated with any decline in accuracy in either the short or long term.

We next examined the total time taken to complete the task (Figure 6(B)). There was no main effect of visit (*F*(1,71) = 0.13, *p* = 0.721), nor was there any significant interaction with visit, indicating that people’s performance did not change from one visit to the next. There was a main effect of session (*F*(1,71) = 7.28, *p* = 0.009): people improved on the second session, which we assume is simply the effect of practice. However, this improvement was affected by TV-group: the passive-3D B-group got worse, while the other two groups improved (session*TV-group: *F*(2,71) = 13.10, *p* < 0.001). This effect also interacted weakly with age-group (session*TV-group*age-group: *F*(6,71) = 2.52, *p* = 0.029). The B group was slightly faster than the other groups on the first test (First Visit/Before), though not significantly so, and there was no main effect of TV-group, indicating that our randomisation was successful. One problem with the coordination data is that not all participants used the same track. However, we repeated the analysis using only the 63 participants who used the ‘medium’-difficulty track on all four occasions (this excluded all under-11s). The interaction with TV-group remained significant, with the B-group again getting worse on average (session*TV-group *F*(2,54) = 7.33, *p* = 0.002).

This raises the question of whether viewing passive (but not active) 3D TV has a short-term effect on coordination. We examined this in a previous paper (Read et al. 2015) and concluded this was not the case. The 116 participants followed up in the present study were a subset of a larger group of 420 participants who were tested on the coordination task before and after watching 2D and 3D TV (Read et al. 2015). In the larger cohort, TV-group had no effect on change in time taken to complete the task (Figure 7 of Read et al. (2015)). Thus, the effect found here did not persist in the larger study. This coupled with the lack of an effect in the active-3D group, or an effect on accuracy as well as time taken, makes it unlikely that these results represent a replicable short-term effect of 3D TV on performance. As far as the main research question addressed in this paper is concerned, we find no evidence for a longer-term effect.

**Figure 7.**
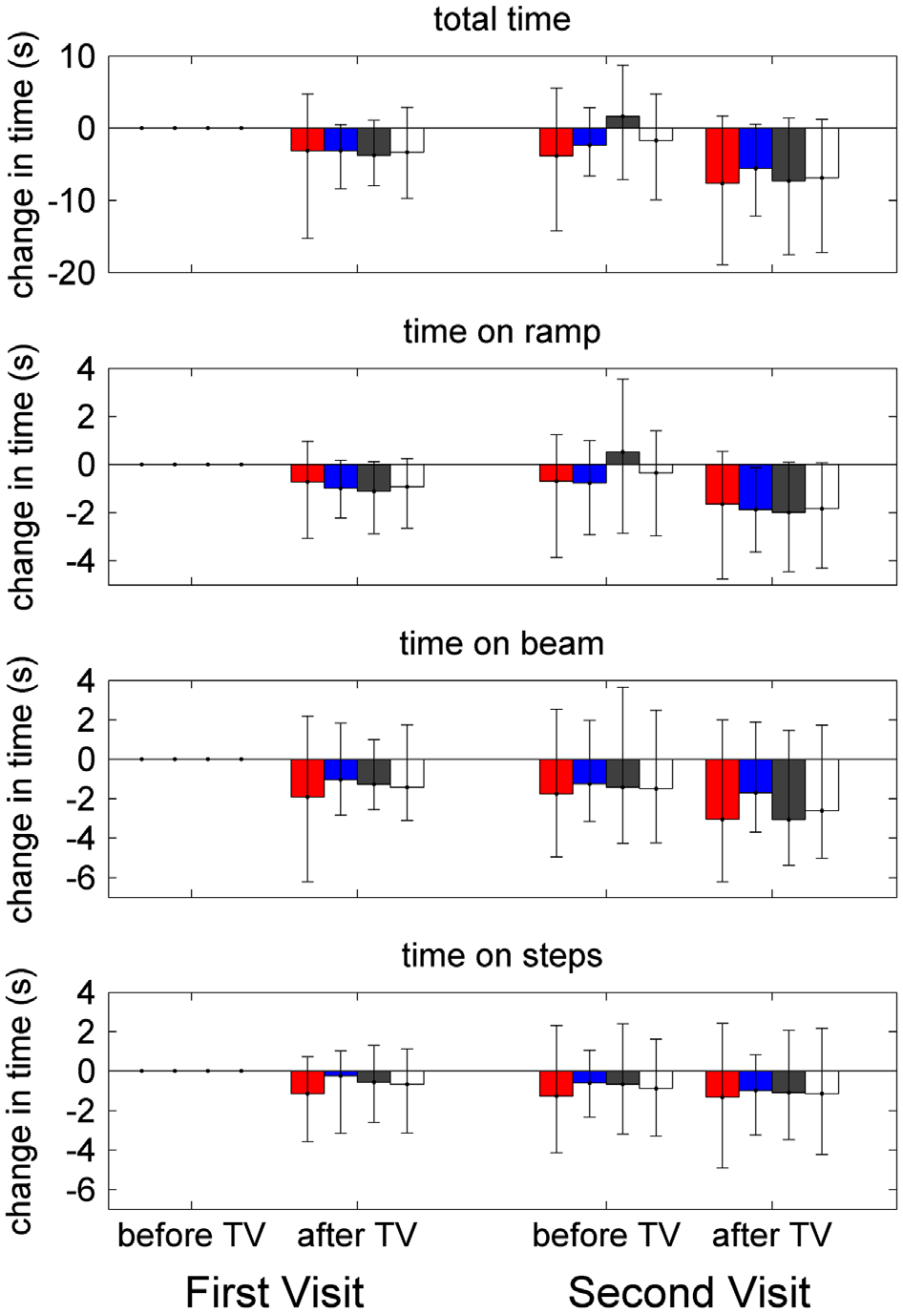
Balance and postural stability.

Notes: How time taken on the balance task varied on the four occasions of testing. Coloured bars show results for the different TV-groups; white bars show results for all participants. Not all data were collected on all occasions for all participants, so N varies slightly between the plots. Data are available for between 107 and 112 participants depending on task/session (40–41 A-group, 36–38 B-group, 31–33 C-group). Height of bars shows mean change in time taken, averaged across participants; error bars run from the 16th to the 84th percentile. As before, data are shown relative to time on First Visit, before TV; these points are therefore zero by definition.

### Balance tests

For the balance tests, participants walked around a simple ‘obstacle course’ consisting of ramps, a balance beam and steps (see Read et al. 2015 for details). We timed participants via pressure-sensitive mats under the flooring material, and also monitored accuracy on the beam and steps tasks. On the beam task, an error consisted of stepping off the beam while attempting to walk along it. On the steps task, participants were asked to step over a set of five obstacles constructed from foam blocks, while not dislodging any of four foam blocks which were balanced in position (Read et al. 2015). Very few participants made such errors; in fact, the median number of errors was zero for all TV-groups on all four test occasions (2 visits, 2 sessions) on both tasks (steps and beam). Thus, it was not possible to analyse the error data meaningfully.

Figure 7 shows the timing data, for both visits and sessions, for the whole task (A) and for each component separately. As in Figure 6, times are shown relative to the time taken on the first occasion of testing to remove between-subjects variation. Once again, participants tend to be faster on the second session of each visit, and on the second visit relative to the first, presumably due to practice/familiarity effects. Once again, we analysed this data using repeated measures ANOVA.

If we look first at total time taken to complete the task, we find a main effect of visit (*F*(1,96) = 5.43, *p* = 0.022) and session (*F*(1,96) = 34.42, *p* < 0.001), reflecting the practice/familiarity effect just noted. Unsurprisingly, there was also a main effect of age, with people between 11 and 25 performing better than those over 25 (*F*(3,96) = 6.29, *p* = 0.001). This was also noted in our larger study on the short-term effects of 3D TV (Read et al. 2015). There was no main effect of TV-group, indicating that our randomisation was successful. Critically, there was also no interaction between TV-group and visit or session, indicating that viewing 3D TV does not affect performance on this task, in either the short (Read et al. 2015) or longer term.

We also analysed the time taken to complete the three different components of the test, the ramp, beam and steps. The results were very similar to those for total time given above. In every case, there was a main effect of visit and age; there was also a main effect of session on ramp and beam but not steps. In no case was there a main effect of TV-group nor, critically, was there an interaction between TV-group and session or visit. This indicates that viewing 3D TV has no effect on performance on this task, either acutely or over the longer term.

### Accelerometry

We monitored two tri-axial accelerometers, one on the hip and one on the chest. Thus, we recorded six accelerations in total, each resulting in a single metric of variability (the SD) during each task. These SDs differed among the six accelerometer axes (*F*(5,68) = 44.30, *p* < 0.001), with the hip sensor showing generally higher SDs than the chest sensor. There was also a difference among the three tasks (*F*(2,71) = 50.10, *p* < 0.001), with SDs being higher on the steps task than the ramp, and higher on the ramp than the beam. The high SDs recorded on the steps task may reflect the high stepping movements required to step over the obstacles, while the low SDs recorded on the beam may reflect the more cautious movements needed on this balance task. Unsurprisingly, the different tasks had different effects on the six different axes (axis*task interaction: *F*(10,63) = 38.95, *p* < 0.001). These results are encouraging because they confirm that our accelerometry measurements are sensitive enough to detect relatively subtle within-subject differences, such as the difference in body motion when walking over a ramp compared when walking along a beam.

**Figure 8.**
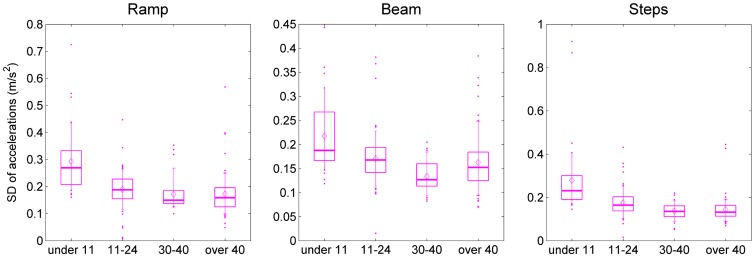
Effect of task on accelerometry.

Notes: Box and whisker plot for the SD of acceleration recorded for the vertical axis of the chest accelerometer (axis 2, Read et al. 2015) during the three different tasks, before viewing TV. Symbols as in Figure 2. For this figure, data have been pooled across participants in all TV-groups and across initial and final visits, since our ANOVA revealed no main effects of these variables. This figure shows that our accelerometry is powerful enough to reveal the effects of age and task.

We were also able to detect the between-subject effect of age. The youngest participants (under 11) showed higher accelerometry SDs than the older ones, with participants in their 30s and 40s having the lowest SDs (*F*(3,72) = 16.24, *p* < 0.001). As might be expected, the age differences affected some accelerometers/axes more than others (age*axis interaction: *F*(15,210) = 3.99, *p* < 0.001). The effect of age was also different for the three different tasks (age*task interaction: *F*(6,144) = 7.23, *p* < 0.001). Some of these effects are visible in Figure 8, which shows the distribution of SDs for the vertical axis of the chest acceleration before TV viewing. This figure shows that SDs are higher for the younger age-groups, especially on the ramp and step tasks. As noted in the previous paragraph, overall, lower SDs were recorded on the beam than on the ramp task. In our oldest age-group, this pattern was reversed, with people over 40 having the lowest variability on the ramp. (Overall, all age-groups recorded the highest SDs on the steps task, although this is not always the case for the particular axis shown in Figure 8.)

We found that the variation in the accelerometry readings increased after they had watched the movie (*F*(1,72) = 7.94, *p* = 0.006), although this did not depend on the type of TV they watched (session*TVGroup interaction: *F*(2,72) = 0.40, *p* = 0.674). Sitting down for 90 min to watch TV did affect the different accelerometry axes differently (session*axis interaction: *F*(5,68) = 4.89, *p* = 0.001).

TV-group A had more variability in their accelerometry readings than the other two groups (*F*(2,72) = 6.34, *p* = 0.003). The differences in variability among the three tasks were also different among the three TV-groups (task*TVGroup interaction: *F*(4,144) = 3.21, *p* = 0.015), but this was a difference of magnitude rather than of which task had the highest or lowest variability. Importantly, none of these effects of TV-group interacted with either session (short-term exposure to S3D TV; see earlier) or visit (longer exposure to S3D TV; visit*TVGroup interaction: *F*(2,72) = 0.59, *p* = 0.559), indicating that the group differences were the result of imperfect randomisation, rather than being an effect of the treatment itself.

**Figure 9.**
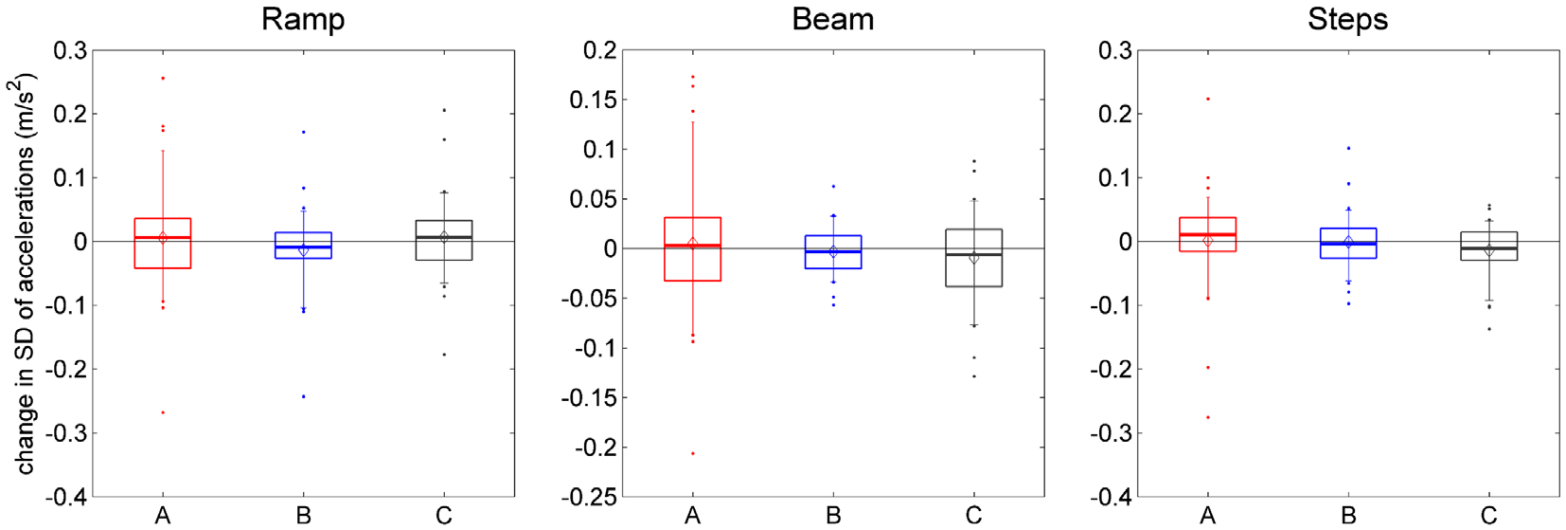
Change in accelerometry across the study.

Notes: Box and whisker plot for the *change* in SD, final visit – initial visit, for the vertical axis of the chest accelerometer during the three different tasks, before viewing TV. Symbols as in Figure 2. For this figure, data have been pooled across participants in all age-groups. The horizontal line shows no change (i.e. SD of this accelerometer/axis/task before TV viewing was the same on both visits). This figure shows that there are no differences between TV-groups.

Figure 9 shows the change between initial and final visits, some 8 weeks apart, for one example accelerometry metric. For each participant for whom both data-sets exist, we took the SD of the vertical acceleration recorded at the chest while the participant carried out the task shown in each panel, before TV viewing. We subtracted this SD at the initial visit from the SD on the final visit, and pooled this change over all participants to obtain the distributions shown in Figure 9. The means and medians are very close to zero, indicating no significant change over the ~8-week period of owning the new TV. Crucially, this was true for participants in all three groups, independently of whether they were given a 2D or 3D TV.

## Power analysis

We estimated that our study has enough power for us to reliably detect an effect of 0.7 of the within-group standard deviations. Given our sample size and the variance in the population,Table 2 gives estimates for the smallest detectable change in various parameters.

**Table 2.** Smallest detectable change in various parameters which we would have detected given our sample size. ‘Change’ refers to the difference across the 8 weeks of the study.

Parameter	Smallest detectable change between visits	Units
Near stereo threshold (Frisby)	28	arcsec
Distance stereo threshold (FD2)	13	arcsec
Binocular visual acuity with best correction at 0.4 m	0.07	logMAR
Binocular visual acuity with best correction at 6 m	0.05	logMAR
Time taken on coordination task, before TV	21	Seconds
Accuracy on coordination task, before TV	14	Percent time buzzing, before TV
Change in time taken on coordination task, before vs. after TV	5.8	% change before vs. after TV
Time taken on ramp task, before TV	2.3	Seconds
Time taken on beam task, before TV	4.4	Seconds
Time taken on steps task, before TV	2.4	Seconds
Accuracy on steps task, before TV	0.5	Number of blocks displaced

## Discussion

This paper reports on a short, preliminary longitudinal study examining the effects of viewing 3D television in the home. We monitored participants’ vision and ocular health, and their performance on visuomotor tasks requiring balance and coordination. There was no evidence of any 3D-related changes over the 2 months of the study.

Our power to detect changes is relatively low. Most of our metrics show relatively wide variability between individuals, while our sample size of 116 individuals, though far larger than previous studies in this area, falls sort of the thousands that would be required to detect subtle changes. For example,Table 2 shows that we would expect to detect only a change in excess of 28 arcsec for stereo thresholds on the Frisby test. This means that if viewing 3D TV somehow damaged stereoacuity, such that over an 8-week time-period stereo thresholds increase by an average of 20 arcsec, we would not have detected this. However, we would have detected an increase of 30 arcsec. This is a relatively large change in stereoacuity. Analysing our wider data-set (Bohr and Read 2013; Read et al. 2015), we estimate that the natural decline in stereoacuity between the ages of about 40 and 60 produces an increase of only around 40 arcsec in mean Frisby threshold. Clearly, therefore, we do not have the power to detect possible small changes in stereoacuity associated with S3D TV. However, our work does place an upper bound on any such changes. Given the current total absence of any such information previously, even this is welcome.

Our work is limited in other respects. Two months is a relatively short time and would not reveal possible developmental changes. It would be valuable to follow up the participants over the next several years and look to see whether any changes emerge over longer timescales. Additionally, participants viewed 3D content *ad libitum*, and most chose to view relatively little: typically less than an hour a day (Figure 2). It therefore remains possible that greater amounts of 3D TV may be associated with problems. Nevertheless, given the concern in the media and the fact that the existing scientific literature (e.g. Hiruma and Fukuda 1993; Yano, Emoto, and Mitsuhashi 2004; Hoffman et al. 2008; Yang and Sheedy 2011; Shibata et al. 2011b; Kane, Held, and Banks 2012; Kim, Kane, and Banks 2012; Kim et al. 2013; Pölönen, Järvenpää, and Bilcu 2013; Kim, Kane, and Banks 2014; Read et al. 2015) consists exclusively of short-term studies examining the impact of viewing stereoscopic 3D for up to 2 h, this study represents a valuable contribution. It is the first study to investigate whether moderate amounts of stereoscopic 3D viewing over a period of several weeks have adverse effects on vision, balance or visuomotor coordination. No such effects were detected.

## Funding

This work was supported by British Sky Broadcasting Ltd; The Royal Society [UF041260].
